# Normal-Pressure Hydrocephalus-Like Appearance in Myotonic Dystrophy Type 1

**DOI:** 10.7759/cureus.53130

**Published:** 2024-01-28

**Authors:** Asuka Suzuki, Koji Hayashi, Yuka Nakaya, Maho Hayashi, Kouji Hayashi, Yasutaka Kobayashi, Mamiko Sato

**Affiliations:** 1 Department of Rehabilitation Medicine, Fukui General Hospital, Fukui, JPN; 2 Department of Internal Medicine, Fukui General Hospital, Fukui, JPN; 3 Graduate School of Health Science, Fukui Health Science University, Fukui, JPN

**Keywords:** mri, brain anatomy, csf tap test, myotonic dystrophy type 1, normal-pressure hydrocephalus

## Abstract

Myotonic dystrophy type 1 (DM1) is one of the monogenic neurological diseases that neurologists most often experience. DM1 can develop several symptoms, including muscle weakness, gait disturbance, urinary incontinence, and cognitive decline. Other hand, normal pressure hydrocephalus (NPH) is more frequent in the elderly population and is characterized by a triad of symptoms, gait disturbance, urinary urge incontinence, and cognitive decline. Therefore, some symptoms overlap between DM1 and NPH. In this report, we described a case of DM1 that presented with a triad of NPH, and NPH-like changes in brain images. A 54-year-old man with DM1 visited our hospital for rehabilitation. He had a history of dyslipidemia, diabetes, and cataracts. He developed muscle weakness, blepharoptosis, and dysarthria at 43 years. Neuro-exam revealed percussion and grip myotonia, distal muscle weakness and atrophy, broad-based gait, and urinary incontinence. The mini-mental state examination score was 18. Brain magnetic resonance imaging revealed enlarged lateral and third ventricles and Evans index was 0.38 (NPH criterion; >0.3), which was mimicking for NPH. Tap test (TT) was evaluated twice. First TT improved clinical symptoms slightly, but second was unremarkable. Based on the second TT result, we could not diagnose with NPH and could prevent unnecessary surgical shunting. Brain imaging of DM1 can show an NPH-like appearance in patients older than 50. Although TT is the gold standard for diagnosing NPH, its sensitivity and specificity vary among reports. TT results should be interpreted with caution before performing a surgical shunt. If necessary, multiple TTs should be considered in DM1 patients.

## Introduction

Myotonic dystrophy type 1 (DM1) is one of the monogenic neurological diseases that neurologists most often experience [1,2]. This cause is an expansion of a CTG triplet repeat in the 3' non-coding region of DMPK, the gene encoding the DM protein kinase [1,2]. DM1 can develop several symptoms [1,2]. In childhood, cognitive and behavioral features are prominent [1,2]. In adulthood, muscular and systemic symptoms can occur including blepharoptosis, wasting of temporalis and masseter, facial weakness, myotonia, distal-dominant muscle weakness and wasting, gait disturbance, diaphragmatic weakness, cataract, central nervous system symptoms like dementia, cardiac diseases like dysrhythmia and atrioventricular block [1,2]. Additionally, diabetes mellitus is a well-known complication of DM1, and if not properly treated, may cause diabetic polyneuropathy [2], leading to exacerbating of gait instability, distal weakness, and neurogenic bladder. In addition, DM1 can develop urological symptoms including urinary incontinence [3,4]. Other hand, normal pressure hydrocephalus (NPH) is more frequent in the elderly population and is characterized by gait disturbance, urinary urge incontinence, and cognitive decline [5]. In a patient with NPH, brain imaging findings are characterized by ventricular enlargement [5]. In this report, we describe a case of DM1 that presented with gait disturbance, cognitive decline, urinary incontinence, and NPH-like changes in brain images.

## Case presentation

**Figure 1:**
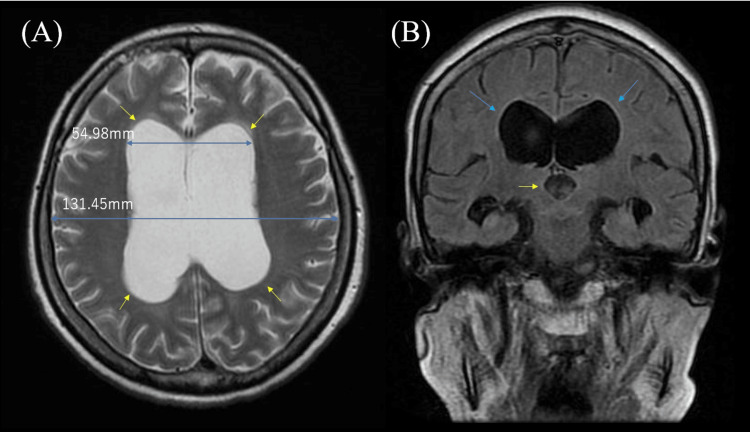
The results of brain MRI. (A) T2-weighted brain MRI showed enlarged lateral ventricles (yellow arrowheads). Evans index (the ratio of the maximum width of the frontal horns of the lateral ventricles and the maximal internal diameter of the skull; blue two-way arrows) was 0.42 (normal-pressure hydrocephalus (NPH) criterion; >0.3). (B) The T2-weighted fluid-attenuated inversion recovery (FLAIR) brain MRI showed enlarged lateral ventricles (blue arrowheads) and third ventricle (yellow arrowhead), but the callosal angle was not narrowed. This patient did not present disproportionately enlarged subarachnoid-space hydrocephalus.

A 54-year-old man with a history of dyslipidemia, diabetes, and cataracts visited our hospital for rehabilitation. At 43 years old, he developed muscle weakness, blepharoptosis, and dysarthria. At 48 years, he was diagnosed with muscular dystrophy type 1 (DM1) by genetic testing. Neuro-exam revealed percussion and grip myotonia, distal muscle weakness and atrophy, broad-based gait, and urinary incontinence. The mini-mental examination score was 18. Blood tests revealed elevated hemoglobin A1c (11.1%). Brain magnetic resonance imaging (MRI) revealed enlarged lateral and third ventricles and Evans index was 0.42 (NPH criterion; >0.3) (Figures 1A, 1B). In our case, neither narrowing of the callosal angle nor disproportionately enlarged subarachnoid space hydrocephalus (DESH) was observed (Figure 1B). Based on his symptoms including gait disturbance, urinary incontinence and cognitive decline, and brain MRI findings of elevated Evans index, we suspected DM1 with NPH. The result of the tap test (TT) showed normal opening pressure of cerebrospinal fluid (CSF) and slightly improved the score of the timed up-and-go (TUG) test and clinical symptoms including gait disturbance. Three months later, we retried the TT. The second TT showed no improvement of TUG or clinical symptoms. On the basis of these findings, we could not diagnose him with NPH.

## Discussion

In this report, we describe a case of DM1 in which complication of NPH was suspected. The patient had symptoms of gait disturbance, cognitive decline, and urinary incontinence. In addition, his brain MRI revealed an enlarged lateral and third ventricle and elevated Evans index. Moreover, the first TT revealed normal CSF pressure, improvement of TUG, and clinical symptoms. These findings seemed to support the diagnosis of NPH. The reason for performing a second TT was that there was insufficient evidence of imaging findings related to NPH such as callosal angle and DESH. In fact, the response to the second TT was poor, and the result of the first TT seemed to be a placebo effect.

DM1 can develop various symptoms throughout the body [1,2]. In addition to the typical symptoms like gait disturbance caused by muscle atrophy and muscle weakness, cognitive decline is a common presentation in DM1 [1,2]. Moreover, urinary symptoms are frequent [3,4]. In pediatric DM1 cases, the most frequently reported urological symptoms were difficulty with toilet training (59.3%), urinary incontinence (22.0 %), enuresis nocturna (10.3%), and voiding (23.5% hesitancy, 4.8% intermittency, and 13.8% dysuria) [3]. Among female DM1 cases, it has been reported that 60% of them developed urinary incontinence [4]. In addition, we thought that functional urinary incontinence is also common in DM1 because muscle weakness and gait disturbance are common. Moreover, one of the common complications of DM1, diabetes can cause polyneuropathy, leading to gait disturbance and urinary incontinence [2]. On the basis of these findings, the symptoms of DM1 resemble a triad of NPH. Therefore, it would be premature to immediately assume DM1 with NPH, even if a triad of NPH is present.

As far as we know, five cases have been reported of myotonic dystrophy (DM) with NPH [6-9]. All five cases were over 50 years old (50-75 years) and had enlarged ventricles or increased the Evans index. Two of the five cases were treated with surgical shunts. Unfortunately, the descriptions of genetic tests for DM and TT for NPH were absent in four of five cases [7-9]. The remaining one case was diagnosed with DM1 by genetic testing, and TT was positive [6]. In addition, coronal imaging of brain MRI or CT was not presented in all five cases. Therefore, the callosal angle has not been evaluated in any case.

In the brain imaging of DM1, several findings are reported including whole-brain atrophy, increased ventricular volume, widespread volume reduction of cortical and deep gray matter and reduced cortical thickness (occipital, parietal and temporal lobes) [10]. Additionally, an MRI study of 112 patients with DM1 revealed that NPH-like appearances including enlarged ventricles and DESH are frequent signs in DM1 over 50 years old [11]. In elderly DM1 group, prevalence of DESH was 21% and the z-Evans index [12], an improved version of the Evans index, increased with age [11]. Notably, there was no significant difference in average callosal angle between DM1 and age-matched control (DM1 and control; 123° vs. 123°) [11]. Additionally, the diagnosis of NPH is generally evaluated by TT, whereas the sensitivity and specificity of TT for NPH vary among previous reports; sensitivity: 72%-100% and specificity: 33%-100% [13-16]. A subsequent study revealed the sensitivity of TT was 71.3% and the specificity was 65% by 100 consecutive cases [17]. Although it varies depending on the literature, what should be noted here is the low specificity of TT. Therefore, it is premature to diagnose NPH even if clinical symptoms and scoring improve after one TT, especially in DM1 cases.

In our case, enlarged ventricles and elevated the Evans index were noted. However, these findings were considered to be due to findings in not NPH but elderly DM1. Furthermore, the callosal angle was normal in our case, which is seemed to be a key finding for distinguishing between DM1 and NPH, considering a previous report. We performed TTs two times, which were also important. Especially in patients with DM1, surgical shunts should be refrained even if a single TT is positive. TT results should be carefully interpreted in DM1 with an NPH-like appearance before surgical intervention.

## Conclusions

To the best of our knowledge, there is no literature highlighting that DM1 resembles NPH in clinical symptoms and brain imaging findings, as well as the importance of interpretation of callosal angle and TT in DM1. In our case, we could determine non-diagnostic for NPH by TT twice, and prevent unnecessary surgical shunting. Considering the variety symptoms of DM1, it is common that a triad of NPH, gait disturbance, urinary incontinence and cognitive decline, appears in DM1 cases. It is common for elderly DM1 patients aged 50 years or older to have an NPH-like appearance including DESH and enlarged ventricles on brain images. The callosal angle is seemed to be a key finding for distinguishing between DM1 and NPH. TT results should be carefully interpreted in DM1 with an NPH-like appearance before surgical intervention. If necessary, multiple TTs should be considered in DM1 patients.
